# β-Cell death is decreased in women with gestational diabetes mellitus

**DOI:** 10.1186/s13098-016-0175-z

**Published:** 2016-08-24

**Authors:** Lauren A. Kenna, John A. Olsen, Michael G. Spelios, Michael S. Radin, Eitan M. Akirav

**Affiliations:** 1 Research Institute, Islet Biology, Winthrop-University Hospital, 101 Mineola Blvd. Rm. 4-39, Mineola, NY 11501 USA; 2 Division of Endocrinology, Diabetes & Metabolism, Winthrop-University Hospital, Mineola, NY USA; 3 Stony Brook University School of Medicine, Stony Brook, NY USA

## Abstract

**Background:**

Gestational diabetes mellitus (GDM) affects approximately 7–17 % of all pregnancies and has been recognized as a significant risk factor to neonatal and maternal health. Postpartum, GDM significantly increases the likelihood of developing type 2 diabetes (T2D). While it is well established that insulin resistance and impaired β-cell function contribute to GDM development, the role of active β-cell loss remains unknown. Differentially methylated circulating free DNA (cfDNA) is a minimally invasive biomarker of β-cell loss in type 1 diabetes mellitus. Here we use cfDNA to examine the levels of β-cell death in women with GDM.

**Methods:**

Second to third-trimester pregnant women with GDM were compared with women with normal pregnancy (PRG), women at postpartum (PP), and non-pregnant (NP) women. Fasting glucose levels, insulin, and C-peptide levels were measured. Serum samples were collected and cfDNA purified and bisulfite treated. Methylation-sensitive probes capable of differentiating between β-cell-derived DNA (demethylated) and non-β-cell-derived DNA (methylated) were used to measure the presence of β-cell loss in the blood.

**Results:**

GDM was associated with elevated fasting glucose levels (GDM = 185.9 ± 5.0 mg/dL) and reduced fasting insulin and c-peptide levels when compared with NP group. Interestingly, β-cell derived insulin DNA levels were significantly lower in women with GDM when compared with PRG, NP, and PP groups (demethylation index: PRG = 7.74 × 10^−3^ ± 3.09 × 10^−3^, GDM = 1.01 × 10^−3^ ± 5.86 × 10^−4^, p < 0.04; NP = 4.53 × 10^−3^ ± 1.62 × 10^−3^, PP = 3.24 × 10^−3^ ± 1.78 × 10^−3^).

**Conclusions:**

These results demonstrate that β-cell death is reduced in women with GDM. This reduction is associated with impaired insulin production and hyperglycemia, suggesting that β-cell death does not contribute to GDM during the 2nd and 3rd trimester of pregnancy.

## Background

Gestational diabetes mellitus (GDM) affects approximately 7 % of all pregnancies in the United States [1, 2], and may complicate as many as 4–5 % of all pregnancies [3], while other estimates from the Hyperglycemia and Adverse Pregnancy Outcomes suggest that as many as 17 % of pregnant women may present with GDM [4]. While transient, GDM has been recognized as a significant risk factor to neonatal health, such as macrosomia and maternal health, including an increased likelihood of Cesarean delivery [5]. Postpartum, GDM significantly increases the likelihood of developing type 2 diabetes (T2D) in the mother [6]. For example, women with GDM show an increase of nearly 7.5 fold of developing T2D when compared to women who had a normal pregnancy [6]. Other studies suggest that GDM may result in abnormal fasting glucose levels in nearly 10 % of women 6 years after delivery [7].

The exact mechanisms responsible for the development of GDM are poorly understood, although insulin resistance and reduced β-cell function both contribute to GDM development [8, 9]. Insulin sensitivity is impaired in women with GDM throughout the pregnancy, with the most significant impairment observed during the *third trimester* of pregnancy [10]. These changes in insulin sensitivity are not fully resolved at postpartum, highlighting the long-term implications of GDM on metabolic control [11]. In addition to changes in insulin sensitivity, β-cell function is also altered. The defect in β-cell function is expressed by a dysregulation of insulin secretion with some groups reporting reduced insulin secretion [11] while others show increased insulin secretion in women with GDM [12]. An elevated ratio of proinsulin to insulin in the blood of women with GDM serves as an additional indicator of β-cell dysfunction in GDM [13].

We have developed the first novel biomarker assay for the detection of β-cell death in diabetes. This assay relies on the measurement of circulating free β-cell derived demethylated free insulin DNA (cfDNA) in the blood [14, 15]. cfDNA shows a good correlation with changes in β-cell mass and insulin content in the pancreas in both animal models of diabetes, as well as in patients with type 1 diabetes and islet transplantation [14, 16]. Here, we apply our biomarker approach for measuring the levels of β-cell derived insulin cfDNA during the 2nd and 3rd trimester of gestation in the blood of women with GDM, women with normal pregnancy, women at postpartum and non-pregnant women. Fasting c-peptide and insulin levels were also measured.

## Methods

### Human subjects

Prospective human serum samples from non-pregnant (NP, n = 10), pregnant (PRG, n = 14), pregnant with gestational diabetes (GDM, n = 22), and postpartum without previous history of diabetes (PP, n = 9) women were obtained. GDM was confirmed by routine oral glucose tolerance test screening (OGTT). Based on previous studies examining the peak of insulin resistance and β-cell dysfunction [10, 11, 13], serum samples were collected during the 2nd and 3rd trimester of pregnancy for both GDM and PRG. Serum samples in the PP group were collected between 3 and 6 weeks postpartum from women with normal pregnancy. All samples were collected over a period of 24 months and stored until analysis. Exclusion criteria included the absence of other major health conditions and the absence of prior history of diabetes (type 1, type 2, and GDM). Patients’ age and average duration of pregnancy were comparable between GDM and NP groups (Table 2). Serum samples were commercially obtained from Analytical Biological Services (ABS) (Wilmington, DE). Samples were collected according to NHS guidelines. Patient consent was obtained prior to participation in the study by ABS.

### DNA collection and bisulfite treatment

DNA was extracted from 300 µL of serum and purified using a DNeasy Blood and Tissue Kit (Qiagen N.V., Valencia, CA) following the manufacturer-recommended protocol. DNA was then subjected to bisulfite treatment and purified on a DNA binding column to remove excessive bisulfite reagent using the EZ DNA Direct Kit (Zymo Research, Irvine, CA).

### Probe-based analysis of circulating demethylated insulin DNA

**Fig. 1:**
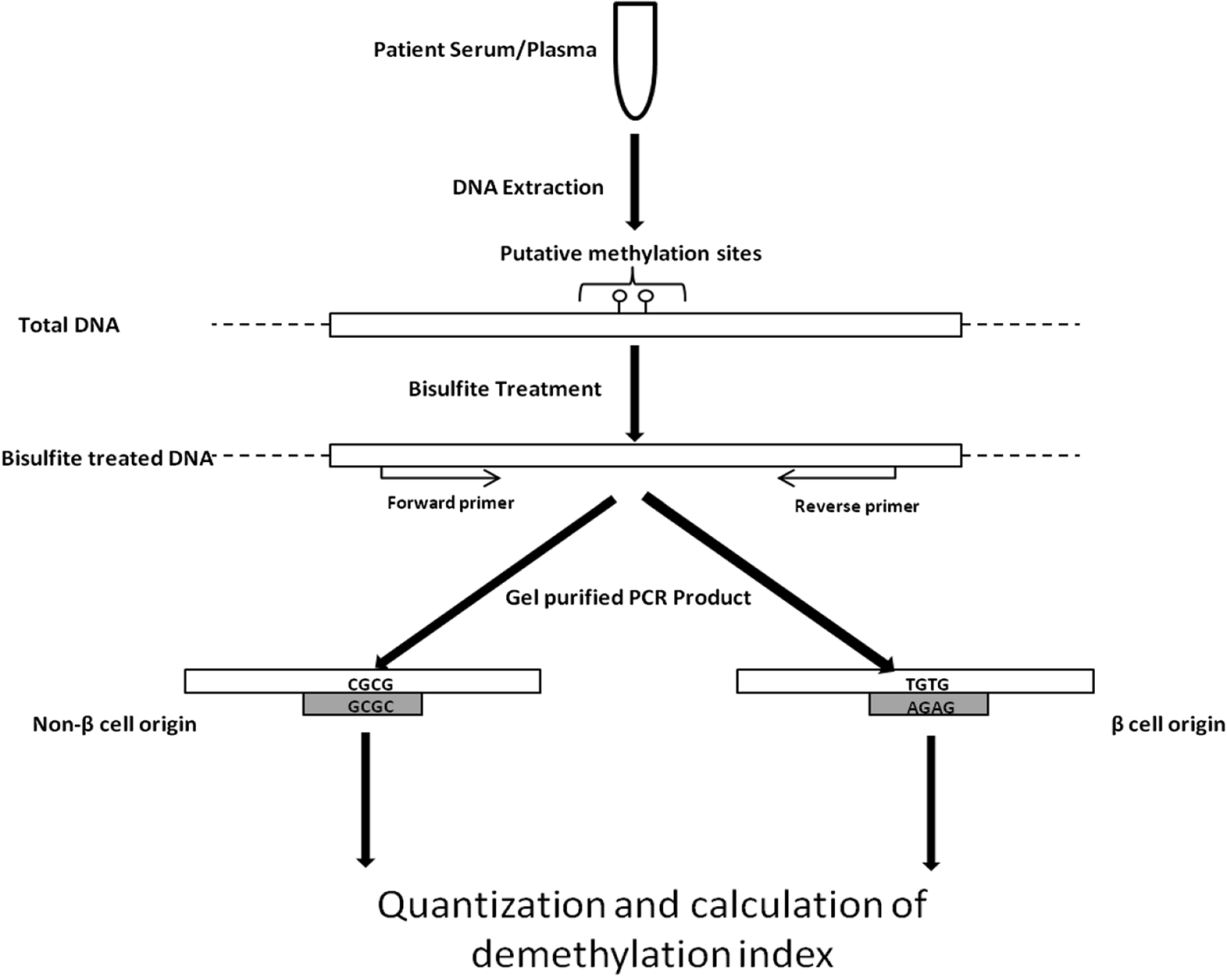
A schematic depiction of β-cell derived insulin DNA biomarker assay. DNA was extracted from 300 μL of serum and converted with bisulfite treatment. Converted DNA was subjected to a first step PCR amplification followed by gel electrophoresis. Gel purified amplicons were tested for the presence of β-cell derived insulin DNA by real-time PCR using methylation-sensitive probes. The relative abundance of demethylated DNA was expressed using the following equation: demethylation index = 2^(methylated cycle number) − (demethylated cycle number)^

Levels of β-cell death were measured as previously described ([14] and Fig. 1). Converted DNA was subjected to a methylation insensitive first step PCR amplification followed by gel electrophoresis to increase template availability and minimize the present impurities. Gel purified amplicons were tested for the presence of β-cell derived insulin DNA by quantitative real-time PCR (qRTPCR) [14]. The relative abundance of demethylated DNA was expressed using the following equation: demethylation index (DMI) = 2^(methylated cycle number) − (demethylated cycle number)^. All samples were analyzed three times by three runs. Interassay variability was corrected using methylated or demethylated plasmids containing the insulin gene. Methylation-sensitive probes were used to detect β-cell derived circulating free DNA. Probe sequences and PCR reaction conditions are described in Table 1.

**Table 1:** Primer and probe sequences and PCR protocols for human insulin analysis

PCR type	Primer designation	Primer sequence 5′ → 3′	Amplicon length	PCR protocol
First-step PCR	Forward	TTAGGGGTTTTAAGGTAGGGTA	295 bp	50 cycles, annealing temperature 57 °C
	Reverse	ACCAAAAACAACAATAAACAATAACTC		
Methylation-specific nested qRTPCR	Common forward	GTGCGGTTTATATTTGGTGGAAGTT		50 cycles, annealing temperature: 52 °C Methylated
	Common reverse	ACAACAATAAACAATTAACTCACCCTACAA		
	Hypomethylated FAM probe	ACCTCCCAACAAATCT		65 °C Hypomethylated
	Methylated FAM probe	TACCTCTCGTCGAATCT		

### Analysis of insulin and c-peptide levels

Fasting insulin and c-peptide levels were analyzed by ELISA [insulin ELISA kit (Millipore Billerica, MA), c-peptide ELISA kit (ALPCO Salem, NH)] on stored serum samples.

### Statistical analysis

Data are expressed as mean ± SEM. The differences between means were analyzed by one-way ANOVA with Tukey’s post hoc test using Prism 5 (GraphPad software). Differences between groups were considered significant at p < 0.05. In some instances groups were analyzed using Student’s t-test. Exact statistical tests used are depicted below.

## Results

### β-cell derived insulin cfDNA levels are decreased in women with GDM

We examined serum samples from a total of 55 women who were non-pregnant (NP, n = 10), pregnant (PRG, n = 14), pregnant with gestational diabetes (GDM, n = 22), and postpartum without previous history of diabetes (PP, n = 9). Table 2 describes subject age and duration of pregnancy (where applicable). Similar age and gestational duration were observed between PRG and GDM (Table 2; subject age: PRG = 33.4 ± 1.5, GDM = 32.9 ± 1.3 years; weeks gestation: PRG = 26.1 ± 1.2, GDM = 25.9 ± 0.4 weeks). Women with GDM were identified by routine OGTT and diabetes was confirmed by measuring fasting blood glucose levels (glucose (mg/dL): GDM = 185.9 ± 5.0).

**Table 2:** Study subject characteristics

Group	N	Age (years)	Week of gestation
NP	10	42.1 ± 2.2*	N/A
PRG	14	33.4 ± 1.5	26.14 ± 1.2
GDM	22	32.9 ± 1.3	25.9 ± 0.4
PP	9	33.4 ± 1.2	4.2 ± 0.6^a^

*NP* non-pregnant, *PRG* normal pregnancy, *GDM* gestational diabetes mellitus, *PP* postpartum

* p < 0.05 vs. all other groups

^a^ weeks post delivery

**Fig. 2:**
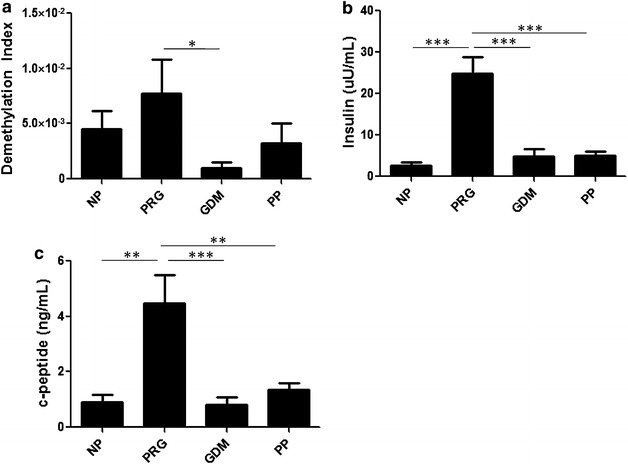
β-cell death is not increased in pregnant women with GDM. Serum samples from NP, PRG, GDM and PP were collected and analyzed as described in the Methods.** a** Demethylation index values of all groups were calculated as described in Methods. ANOVA p = 0.05; Tukey’s MCT PRG vs. GDM *p < 0.05.** b** Random fasting insulin levels in all groups. ANOVA p < 0.0001; Tukey’s MCT ***p < 0.001.** c** Random fasting C-peptide levels in all groups. ANOVA p < 0.0001; Tukey’s MCT **p < 0.01; ***p < 0.001

qRTPCR of insulin DNA in PRG and GDM groups showed a significant ~7.5-fold decrease in β cell loss in GDM (Fig. 2a; Demethylation index: PRG = 7.74 × 10^−3^ ± 3.09 × 10^−3^, GDM = 1.01 × 10^−3^ ± 5.86 × 10^−4^, ANOVA, p < 0.05), while DMI levels did not differ between PRG and NP women (PRG = 7.74 × 10^−3^ ± 3.09 × 10^−3^, NP = 4.53 × 10^−3^ ± 1.62 × 10^−3^, NP vs. PRG). Approximately 4 weeks following delivery, PP group showed levels comparable with NP women (NP = 4.53 × 10^−3^ ± 1.62 × 10^−3^, PP = 3.24 × 10^−3^ ± 1.78 × 10^−3^). These data suggest that β-cell death does not increase in women with GDM when compared to women with normal pregnancy.

### Fasting insulin and c-peptide levels are decreased in the blood of women with GDM

Our inability to detect elevated β-cell derived cfDNA in the blood of women with GDM prompted us to examine the levels of insulin and c-peptide in these women. In our study, both fasting insulin and c-peptide levels were significantly decreased in GDM when compared with PRG, with the highest overall levels measured in the PRG group (Fig. 2b Insulin: PRG = 24.82 ± 4.09, GDM = 4.91 ± 1.65, PP = 5.03 ± 0.92, and NP = 2.64 ± 0.69 μU/mL; ANOVA p < 0.0001, PRG vs. GDM p < 0.001, PRG vs. PP p < 0.001, PRG vs. NP p < 0.001. Figure 2c c-peptide: PRG = 4.48 ± 1.03, GDM = 0.82 ± 0.25, PP = 1.36 ± 0.22, and NP = 0.92 ± 0.26 ng/mL; ANOVA p < 0.0001, PRG vs. GDM p < 0.001, PRG vs. PP p < 0.01, PRG vs. NP p < 0.01). This reduction in β-cell function without an increase in β-cell loss further supports the conclusion that β-cell death does not increase under the condition of GDM, and that reduced β-cell function and/or increased insulin resistance are most likely the principal causes for the development of GDM.

## Discussion

In GDM, the ability of β-cells to control glucose levels is impaired due to an overall insulin resistance, increased insulin demand, and impaired β-cell function [9]. Nikolic et al., have recently reported the diminished levels of incretins, such as glucagon-like peptide 1 (GLP-1) and glucose-dependent insulinotropic peptide (GIP), may strongly contribute to development of GDM [17]. Other abnormalities, such as high levels of small dense low-densitiy lipoprotein in women with GDM may also contribute to the impaired metabolic control in these patients [18]. GDM can increase the likelihood of developing T2D or impaired glucose tolerance postpartum [6, 7].

Several factor such as the steroid hormones, progesterone and estrogens have been implicated in β-cell function as they can directly affect insulin secretion and β-cell proliferation [19, 20]. Moreover, progesterone’s putative role in promoting β-cell death by apoptosis may also add an additional stress on β-cell mass in GDM [21, 22]. In this report, we used a novel method for the detection of β-cell death to determine the β-cell loss in GDM by measuring the levels of demethylated insulin cfDNA in the blood of women with GDM [14]. We show that β-cell death did not increase in women with GDM during the 2nd and 3rd trimester when compared with normal pregnancy or with non-pregnant women. In fact, overall β-cell turnover was reduced when compared with normal pregnancy, suggesting that natural β-cell turnover is slowed down in these patients to compensate for the need for higher insulin levels. In our cohorts, women with GDM had impaired β-cell function as measured by an overall reduction in fasting insulin and c-peptide levels.

Xiang et al. previously showed that impaired regulation of glucose clearance, glucose production, and reduced β-cell function are all found in late stage GDM [23]. Recent studies examining β-cell function in Asian women provide additional support to the notion that β-cell function and glucose tolerance are decreased in GDM when compared with normal pregnancy [9]. This deterioration in β-cell function was most evident during late pregnancy. Our measurement of fasting insulin and c-peptide levels in women with GDM and normal pregnancy revealed a marked reduction in the levels of both hormones in GDM when compared with normal pregnancy. In fact, both c-peptide and insulin in GDM were at levels similar to those found in non-pregnant women and women at PP suggesting an inability of the pancreas to respond to the increased metabolic demand during pregnancy. Alterations in insulin production in GDM have been previously documented, although with contradicting results. Xiang et al. and Kautzky-Willer et al. showed an overall increase in insulin levels during steady-state condition in women with GDM [12, 23], while Hamko et al. showed a decrease in insulin levels in women with GDM when compared with controls [11]. Our data support the latter findings, and may result from the fact that serum samples were collected under fasting conditions.

The lower levels of β-cell derived DNA in women with GDM may represent a mechanism by which β-cell mass is preserved under the conditions of increased metabolic demand and poor glycemic control. Indeed, animal studies examining the effects of pregnancy on β-cell mass are marked by a high proliferation rate of β-cells and an overall increase in β-cell mass prior to delivery [24]. Furthermore, the fact that β-cell function is altered with age [25] suggests that β-cell turnover may be different in younger women with GDM when compared with our cohort (both GDM and PRG were in their early 30s). Future studies examining women with GDM at various ages may better address the effect of age on β-cell loss and pregnancy.

The findings presented above suggest a minimal role for β-cell death in women with GDM during late pregnancy. This interesting finding supports the current notion that insulin resistance, glucose intolerance, and β-cell dysfunction are the primary contributors to the development of GDM. In postpartum, β-cell death is reduced and reaches levels seen in non-pregnant women. This data contradicts previous findings in the rat showing increase apoptosis in females post partum as early as 4 days PP with levels returning to normal at 10 days PP [26]. This discrepancy between rodent and human data may stem from the fact that sera samples collected from PP women were obtained at ~4 weeks post delivery, raising the possibility that β-cell contraction may occur earlier (for example during the 3rd trimester or immediately following delivery). Recent reports suggest a persistent defect in β-cell function and the development of T2D in women with a history of GDM [6, 27]. This implicates that β-cell function and β-cell mass are affected PP in women with GDM. Future studies including larger patient cohorts at various gestational stages and in women with previous incidents of GDM may shed new light on the underlying mechanisms that lead to the increased risk of developing type 2 diabetes.
